# Predictors of mortality and rebleeding in acute upper gastrointestinal bleeding: A prospective hospital-based study

**DOI:** 10.6026/973206300221109

**Published:** 2026-02-28

**Authors:** Shrey Singh, Mohamad Akram, Saurabh Singh

**Affiliations:** 1 Department of Medicine, Himalayan Institute of Medical Sciences, Swami Rama Himalayan University, Jolly Grant, Dehradun, India

**Keywords:** Gastrointestinal hemorrhage, peptic ulcer hemorrhage, esophageal varices, risk factors, hospital mortality, rebleeding

## Abstract

Acute upper gastrointestinal bleeding is a major emergency cause of transfusion and in hospital death but local data on mortality and rebleeding predictors is limited. This prospective observational study included 138 adults, most were males 66.7% and age 31-50 years 40.6% with frequent alcohol use 43.5% and NSAID exposure 36.2%. Peptic ulcer disease was the commonest cause 56.5% followed by variceal bleed 25.4% and severe anaemia Hb <7 g/dL was present in 29% with transfusion required in 63.8%. Endoscopic hemostasis was achieved in 87% but rebleeding occurred in 13% and mortality was 9.4% with variceal bleed contributing most rebleeds and peptic ulcer disease accounting for most deaths, so early risk scoring fast endoscopy and correction of reversible host factors remains important.

## Background:

Upper gastrointestinal bleeding is bleeding proximal to ligament of Treitz and usually presents with hematemesis melena or both [1]. In Indian setting alcohol use NSAID exposure and chronic liver disease are common and endoscopic patterns may vary with referral load and hospital level [2]. Male predominance was seen across the included COVID-GIB cohorts and the Indian UGIB series within this review reported 70.8% males [3]. Shenoy *et al.* reported higher mortality with older age low hemoglobin and hemodynamic instability [4]. During COVID period Merza *et al.* reported higher UGIB mortality suggesting access delay and drug exposure as possible contributors [5]. Risk stratification at admission remains practical and Pemmada *et al.* reported ABC score performed better for 30 day mortality than Rockall and Glasgow Blatchford scores supporting early bedside scoring in ED triage [6]. Therefore, it is of interest to evaluate predictors of rebleeding and mortality among acute UGIB patients in our tertiary care setting to improve early recognition and management.

## Materials and Methods:

This was a hospital based prospective observational study done in Department of Medicine of a tertiary care teaching hospital over 18 months. IEC approval was obtained and written informed consent was taken from patient or attendant before inclusion. Adults aged 18 years and above presenting to emergency department or medicine ward with features of UGIB were included. UGIB was defined as hematemesis coffee ground vomiting or melena with endoscopic evidence of bleeding proximal to ligament of Treitz. Lower GI bleed cases were excluded. Patients with bleeding disorders recent trauma or refusal of consent were excluded. History covered age sex comorbidities alcohol intake NSAID use prior peptic ulcer or variceal bleed and chronic liver disease. Admission parameters recorded were pulse blood pressure pallor and shock. Investigations included CBC LFT RFT serum albumin coagulation profile and random blood sugar. Endoscopy was done after hemodynamic stabilisation and source was labelled as variceal or non variceal. Transfusion requirement number of units and hospital stay were recorded. Patients were followed till discharge or death and outcome was survived died or discharge against medical advice. Predictors assessed were age hypotension hemoglobin <7 g/dL need for more than 3 transfusions hypoalbuminemia comorbidities and major baseline risks. Data entry was done in Microsoft Excel and analysis was done in SPSS version 26. Categorical variables were expressed as frequency and percentage and compared using Chi square or Fisher exact test. Continuous variables were compared using independent t test and p value less than 0.05 was taken as significant.

## Results:

In 138 adults with UGIB, baseline profile showed age 31-50 years in 40.6% with male predominance 66.7% and low socioeconomic status 43.5% with common exposures to alcohol 43.5% and NSAIDs 36.2% and comorbidities mainly hypertension 32.6% chronic liver disease 29.0% and diabetes 25.4% (Table 1). Peptic ulcer disease was the leading etiology 56.5% followed by variceal bleed 25.4% Mallory-Weiss tear 8.7% erosive gastritis 7.2% with malignancy and normal endoscopy each 2.2% (Table 2). Severe anaemia Hb <7 g/dL was present in 29.0% and transfusion was required in 63.8% with 1-2 units in 43.5% and almost all received PPI 94.2% while octreotide was used in 32.6% mainly for variceal bleed and H. pylori positivity was 47.1% (Table 3). Rockall score was low in 43.5% intermediate in 39.9% and high in 16.6% and endoscopic hemostasis was achieved in 87.0% with rebleeding in 13.0% and hospital stay 3-7 days in 54.3% and more than 7 days in 16.7% and overall mortality 9.4% (Table 4). Among rebleeding cases n=18 variceal bleed was most frequent 55.6% and among deaths n=13 peptic ulcer disease contributed most 61.5% and no deaths occurred in Mallory-Weiss tear or erosive gastritis (Table 5).

## Discussion:

Our cohort shows UGIB is still high acuity with male predominance and clinically important anaemia with transfusion need. An ED based cohort also reported melena as commonest presentation with high PRBC transfusion need and meaningful in hospital mortality so UGIB remains dangerous even before endotherapy [7]. In our dataset variceal bleed stayed important but not dominant which suggests mixed epidemiology and this depends on centre case mix. An Indian tertiary emergency dataset reported variceal bleeds as majority with alcohol related cirrhosis as main driver showing how local liver disease burden shifts the etiologic split [8]. Outcome is driven by severity and host reserve more than lesion label and a prospective emergency study showed comorbidity and prior liver disease increased adverse outcomes which fit our pattern [9]. Global peptic ulcer estimates show rates may fall but absolute case load stays high due to ageing and population size so ulcer bleed burden will continue in South Asia [10]. Our lesion mix ulcer varices Mallory Weiss erosive are similar to Indian endoscopy audits where varices and duodenal or gastric ulcers lead and alcohol and NSAID exposure remain common background risks [11]. Length of stay and downstream outcomes depend on comorbidity and medication burden and modelling studies show antithrombotic use and higher diagnostic burden prolong stay so UGIB care needs discharge planning and complication prevention early [12]. For triage we used bedside scores and there is emerging value of simple blood indices and comparative work shows AIMS65 Rockall GBS have moderate mortality discrimination and HALP also had usable AUC suggesting added value when endoscopy access or ICU beds are tight [13]. HALP plausibly reflects inflammation anaemia and nutrition and lower HALP predicted mortality in acute MI cohorts and in sepsis it showed independent short term mortality prediction which supports its role as a low reserve marker relevant to high risk UGIB [14, 15]. UGIB can tip into systemic collapse and large in hospital cardiac arrest data links GI bleeding with higher mortality and longer stay [16]. Elderly non variceal ulcer bleeds show mortality clustering with higher ASA or Rockall and lower albumin or haemoglobin which matches the frailty biology we see clinically [17]. Score comparisons across settings suggest no single score is perfect but AIMS65 GBS and Rockall remain practical for early escalation with early resuscitation early scope and parallel host factor correction [18]. In our cohort haemostasis was achieved in most cases but rebleed and deaths clustered in a small severe subgroup with variceal bleeds recurring more and ulcer bleeds contributing more to mortality. This work adds local prospective data linking exposure profile lesion pattern and bedside score strata with rebleeding and in hospital death and it highlights a small actionable high risk group where fast endoscopy and host factor correction can change outcome.

## Conclusion:

Upper gastrointestinal bleeding mainly affected middle-aged males from lower socioeconomic groups. Peptic ulcer disease was the leading cause, with variceal bleeding emerging due to rising liver disease. Low hemoglobin, high Rockall score and comorbidities predicted poor outcomes. Early endoscopy achieved good hemostasis, but mortality remained significant in high-risk patients. Combining simple lab-based indices like HALP with clinical scores may improve early prognosis and guide timely management.

## Figures and Tables

**Table 1:** Sociodemographic and clinical profile of patients

**Variable**	**Category**	**Number (n)**	**Percentage (%)**
Age group (years)	18-30	32	23.2
	31-50	56	40.6
	>50	50	36.2
Gender	Male	92	66.7
	Female	46	33.3
Education	Illiterate	28	20.3
	Primary	45	32.6
	Secondary	40	29
	Graduate	25	18.1
Socio-economic status	Lower	60	43.5
	Middle	55	39.9
	Upper	23	16.6
Comorbidities	Hypertension	45	32.6
	Diabetes mellitus	35	25.4
	Chronic liver disease	40	29
	None	18	13
Habits	Alcohol use	60	43.5
	NSAID use	50	36.2

**Table 2:** Etiological and endoscopic findings

**Etiology / Endoscopic finding**	**Number (n)**	**Percentage (%)**
Peptic ulcer disease	78	56.5
Variceal bleeding	35	25.4
Mallory-Weiss tear	12	8.7
Erosive gastritis	10	7.2
Malignancy	3	2.2
Normal endoscopy	3	2.2

**Table 3:** Laboratory parameters and treatment measures

**Parameter or Management**	**Category**	**Number (n)**	**Percentage (%)**
Hemoglobin (g/dL)	<7	40	29
	7-10	65	47.1
	>10	33	23.9
Blood transfusion units	0	50	36.2
	1-2	60	43.5
	>2	28	20.3
Drug usage	PPI given	130	94.2
	Octreotide given	45	32.6
H. pylori status	Positive	65	47.1
	Negative	73	52.9

**Table 4:** Risk stratification and outcome profiles

**Variable**	**Category**	**Number (n)**	**Percentage (%)**
Rockall score	Low (0-2)	60	43.5
	Intermediate (3-5)	55	39.9
	High (>5)	23	16.6
Endoscopic outcome	Hemostasis achieved	120	87
	Rebleeding	18	13
Hospital stay (days)	<3	40	29
	3-7	75	54.3
	>7	23	16.7
Overall outcome	Survived	125	90.6
	Died	13	9.4

**Table 5:** Etiology wise distribution of rebleeding and mortality

**Factors**	**Rebleeding (n = 18)**	**Percentage (%)**	**Mortality (n = 13)**	**Percentage (%)**	**p-value**
Variceal bleeding	10	55.6	5	38.5	0.001
Peptic ulcer disease	5	27.8	8	61.5	
Mallory-Weiss tear	3	16.6	0	0	
Erosive gastritis	0	0	0	0	
